# Overfitting by design: neural network density functionals for water

**DOI:** 10.1038/s41524-026-02224-5

**Published:** 2026-07-16

**Authors:** Karim K. Alaa El-Din, Antonius von Strachwitz, Ana Coutinho Dutra, Sam M. Vinko

**Affiliations:** Department of Physics, University of Oxford, Oxford, UK

**Keywords:** Chemistry, Materials science, Mathematics and computing, Physics

## Abstract

In density functional theory, simpler exchange-correlation (XC) approximations such as the local density approximation (LDA) are favored for computational speed but rely on limited information, leading to a trade-off between accuracy and generality. Machine-learned XC approximations have seen a lot of interest to address this problem. Here, we train a neural network LDA using a differentiable Kohn-Sham solver, imparting system-specific expertise for water and sacrificing generality for accuracy. Our model achieves 1 kcal / mol errors on gold standard coupled cluster ionization and atomization energies, and improves predictions of spectral lines, electron density distribution, and equilibrium geometry from as few as eight configurations used for training. We proceed to perform transfer learning and obtain results comparable to higher-rung PBE and B3LYP functionals on the WATER27 subset of the GMTKN55 database, even when only a single two-molecule binding energy is used in the transfer process. We furthermore assess performance of this model on a variety of other prediction tasks, including forces, dipole moments, and energetic ordering of isomers. Overall, we demonstrate that simple specialist functionals can be trained from little data, and that transfer learning can aid in this process. By enhancing predictions while maintaining low training costs, we make training of specialist functionals on different systems more accessible. Our approach of training a modified XC density functional approximation (DFA) furthermore allows for a highly interpretable result, as the neural network directly corresponds to a correction of the XC energy per electron.

## Introduction

In the sixty years since its original inception, density functional theory (DFT) has seen an astonishing range of applications^1–3^, driven by the high accuracy at relatively low cost inherent to this framework^4^. From modeling planetary interiors^5^ to hydrogen storage^6^ and chemical receptor binding studies^7^, few tools have proven as influential as DFT in physics, material science, and beyond. Thus, there is a sustained interest in possible improvements in accuracy or computational speed^8,9^, which could open the door to novel applications, and accelerate existing use cases of DFT.

Much of this interest has focused on the exchange-correlation (XC) functional, which encodes the many-body effects of an interacting Coulomb system. In principle, a universal XC term exists but it cannot be computed efficiently^10^ and in practice, a range of density functional approximations (DFAs) are used instead^11^. These range from purely local approximations^12^ to semi-local^13^ and hybrid approaches^14,15^ which incorporate additional information and correspondingly tend to be both more accurate and expensive. Collectively, these approximations are commonly referred to as rungs on Jacob’s ladder^16^, with each rung containing different DFAs utilizing the same amount of input information. In total, there are over 700 DFAs reported in the library of XC functionals (LibXC)^17^, with each corresponding to a different parametrization of the XC term. Such parameterizations may be guided either by non-empirical derivations, or empirical fitting processes.

Recently, machine-learned DFAs have seen a surge of interest^18–21^, but have failed to see broad adoption despite the promising results they deliver^22–26^. In many cases, these approaches have been employed to find a better approximation to the universal XC functional at a given rung of Jacob’s ladder. In other words, the goal is to advance the Pareto front across all of chemical space without increasing the complexity of the DFA parameterization. However, it is unclear whether such a general advancement is possible at each rung, especially for the non-hybrid semi-local rungs constituted by local density approximations (LDAs), generalized gradient approximations (GGAs), and Meta-GGAs. The fact that no model has replaced the Perdew-Burke-Enzerhofer (PBE) GGA^27^ and Perdew-Wang (PW) LDA^12^ on their respective rungs, despite concerted efforts to find more effective models across the past three decades, would suggest that any improvement across all of chemical space, if possible, would be small. Nevertheless, the popularity of these lower-rung models clearly highlights the importance of keeping computations fast and scaling favorably, particularly for modeling larger systems.

We show that improvements in accuracy can be made in this regime from very little data by sacrificing generalizability across chemical space, if an appropriate approach is chosen. To this end, we leverage a differentiable Kohn-Sham solver and the surrogate training embedded in physics (STEP) paradigm^28^. This extends recent work by von Strachwitz et al.^29^, demonstrating the usage of STEP-DFT to reconstruct known functionals from little data. While system-specific ML-DFAs have been proposed in the past^30,31^, the requirements for large datasets typically make them unsuitable as an approach to arbitrary systems. By combining this idea with a very simple functional form and an adjoint solver for regularization, we highlight that this can be done with very sparse datasets. We develop a specialist density functional approximation to characterize both single- and multi-molecule interactions in water, a system both ubiquitous and notoriously challenging to model due to the interplay of strong hydrogen bonds and weak van der Waals interaction. First, we construct a neural LDA trained on single-molecule properties of water obtained from only eight coupled cluster with singlet, doublet, and pertubertive triplet (CCSD(T)) calculations, and demonstrate that the training properties can be learned to within 1 kcal / mol across a wide range of configurations, while simultaneously optimizing the electronic density of the system. We selected 1 kcal / mol as the desirable error level, as this constitutes chemical accuracy when comparisons are made against gold-standard calculations. In addition, we find that our trained DFA reproduces the correct density with much higher accuracy than other, higher-rung DFAs, indicating that both density-driven and functional driven errors are addressed. Note that these gains are due to the restrictions in chemical space to which the model is applicable, and thus constitute a departure from the uniform electron gas limit which typically serves as the justification for LDA models. Our work constitutes a purely heuristic set of models. We then proceed to consider multi-molecule systems from the WATER27 subset of the GMTKN55 database, and apply transfer learning from the single-molecule model in combination with a single scalar CCSD(T) two body energy to realize large gains in performance across the entire dataset containing clusters of up to 20 molecules. We demonstrate that these gains persist in the complete basis set (CBS) limit, and that pre-training on single-molecule is required to realize them. We furthermore show that the single-molecule model shows no significant decrease in performance on the WATER27 dataset compared to the baseline PW-LDA, indicating that even outside their narrow training domain, specialist DFAs may remain suitable.

Although a growing body of similar efforts exists, there are key differences between our approach and other work published to date. First, our use of a small, simple feed-forward neural network as an additive correction to the XC term with the same input parametrization greatly reduces the complexity of the DFA compared to Kirkpatrick et al.^25^, who rely on inclusion of Hartree-Fock energy densities and use a much larger dataset to construct a generalist DFA. For models which focus on water, Dick et al.^32^ use a construction based on spatial projectors and a PBE base model to demonstrate data efficiency on single molecules, and rely on much larger datasets containing dimers and trimers to extend predictions to water clusters. Bogojeski et al.^33^ restrict themselves to single molecule systems, and only consider total energies of the system for evaluation, although they similarly focus on smaller training sets. On the other hand, Fritz et al.^30^ start from a van der Waals DFA which includes non-local interactions and use Bayesian optimization to fit a large dataset of dimer and trimer interaction energies in water. Finally, DeePKS, proposed by Liang et al.^31^ has been shown to obtain strong results on water systems using an adjoint method differentiable KS solver, although they also rely on much larger datasets. From a more generalist perspective, Kasim & Vinko^23^ built a model investigating only a few specific properties across a large range of chemical space, while Li et al.^24^ restrict themselves to hydrogen in one dimension. Overall, the simplicity of our model and small dataset required for effective training of a specialist DFA distinguish this work from previous research. Finally, machine learned potentials provide a different avenue to enhanced predictions of molecular structure, particularly in a msolecular dynamics context^34,35^. However, they also require large datasets for training and are usually bound by the quality of their training data derived from DFT, which can be a limiting factor^36^. Thus, better DFAs are of interest also to the machine learning potential community, as they can improve training data for potentials.

## Results

### Training neural density functional approximations

**Fig. 1:**
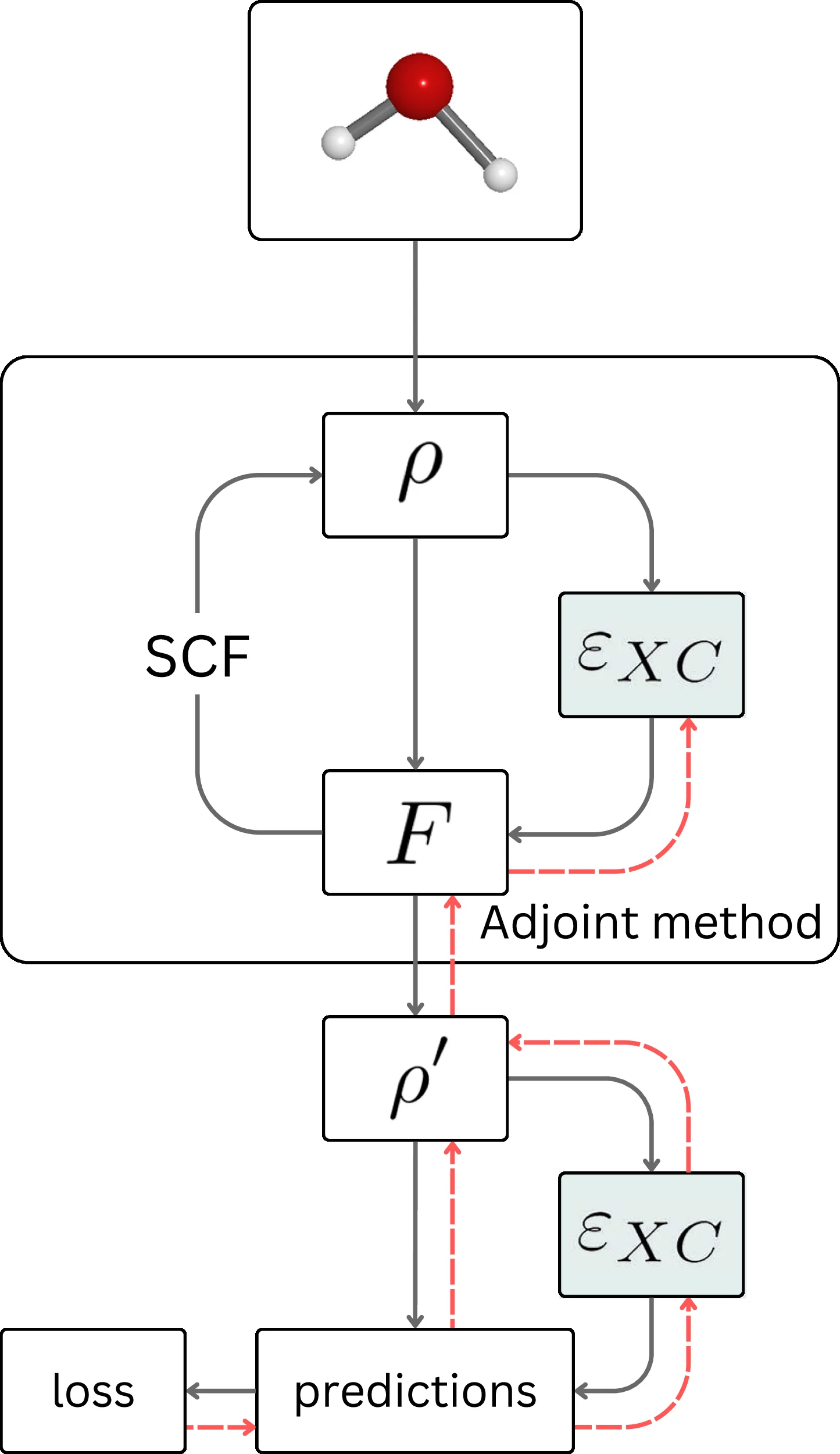
The algorithm used to train DFAs within a Kohn-Sham solver, as implemented by Kasim et al.^37^. Black arrows indicate forward passes while red ones indicate backpropagation. *ϵ*_*XC*_ denotes the DFA which is optimized in this work.

To perform training of the neural DFA, we utilize the differentiable quantum chemistry (DQC) code^37^. This implementation of Kohn-Sham DFT is fully differentiable and makes use of the adjoint method for computational efficacy, as illustrated in Fig. 1. We use a self-consistent field (SCF) solver to obtain converged estimates of the Fock matrix *F* and the electron density *ρ*, from which our objectives are derived as detailed in section 1. Errors of these predictions may then be propagated backwards as losses, to obtain a corresponding error on the electron density and Fock matrix. While the exchange-correlation term contributes to these converged properties at every step of the SCF loop, we can leverage adjoint calculations as outlined in Kasim et al.^38^ to obtain the derivative of the converged Fock matrix with respect to the XC term. Overall, this greatly improves performance and allows for a dynamic and efficient Kohn-Sham solver design, without considerations for backpropagation through the loop.

We follow this design to train a neural field correction to an existing DFA at the relevant level of Jacob’s ladder, the details of which can be found in section 1. For the actual training cycle, we perform the KS calculation for each system at each epoch, backpropagating errors to update the DFA step by step. While expensive, this process is not prohibitive if training on small systems like those used here, and allows for direct optimization of the DFA with respect to objectives of interest.

### Application to single molecules of water

We begin by training on the H_2_O geometries contained within the ANI1-ccx dataset^39^, details and a visual summary of which can be found in the supplementary material. In-house CCSD(T) calculations were at the pc-2 basis level and used to generate labels for atomization, ionization, and total energies, as well as a localized energy loss (LEL) calculated from the electron density obtained via the CCSD(T) reduced density matrix as

1 $${{\rm{LEL}}}=\sum \limits_{i=1}^{N}| {\varepsilon }_{X}({\rho }_{i})-{\varepsilon }_{X}({\tilde{\rho }}_{i})| {V}_{i}.$$

Here, *ε*_*X*_ is the well-known Slater exchange approximation, *i* indexes the volume elements of the molecular grid, and *V*_*i*_, *ρ*_*i*_ correspond to the volume and electron density at the grid point respectively. Overall, this definition of LEL yields a non-unique volume weighted mean absolute error of the electron density adjusted for the exchange energy contributed by a given density. The LEL is useful insofar as it ensures consistent scale between different error terms and avoids ad hoc weighting corrections. It is not to be confused with the LES established in the literature^40^, which has a different definition, although both ensure locally correct behavior in addition to molecular energetics.

**Fig. 2:**
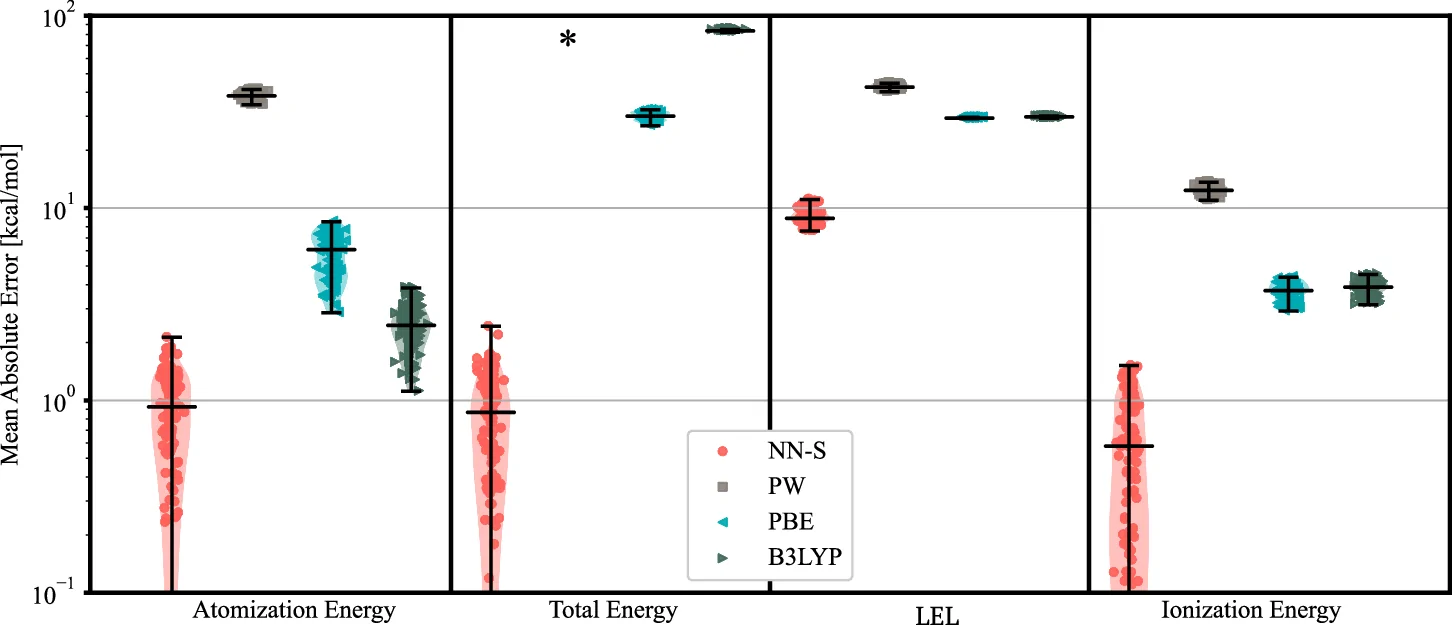
The absolute errors on the testing set of single molecule systems for different models. Note that the NN-LDA achieves errors below 1 kcal / mol on the three energy properties (atomization, total, and ionization energies), while performing best on the localized energy loss (LEL) as defined in Eq. (1). The total energies for PW-LDA are omitted, as all errors are in excess of 100 kcal/mol. This is indicated with a black asterisk in the figure.

**Fig. 3:**
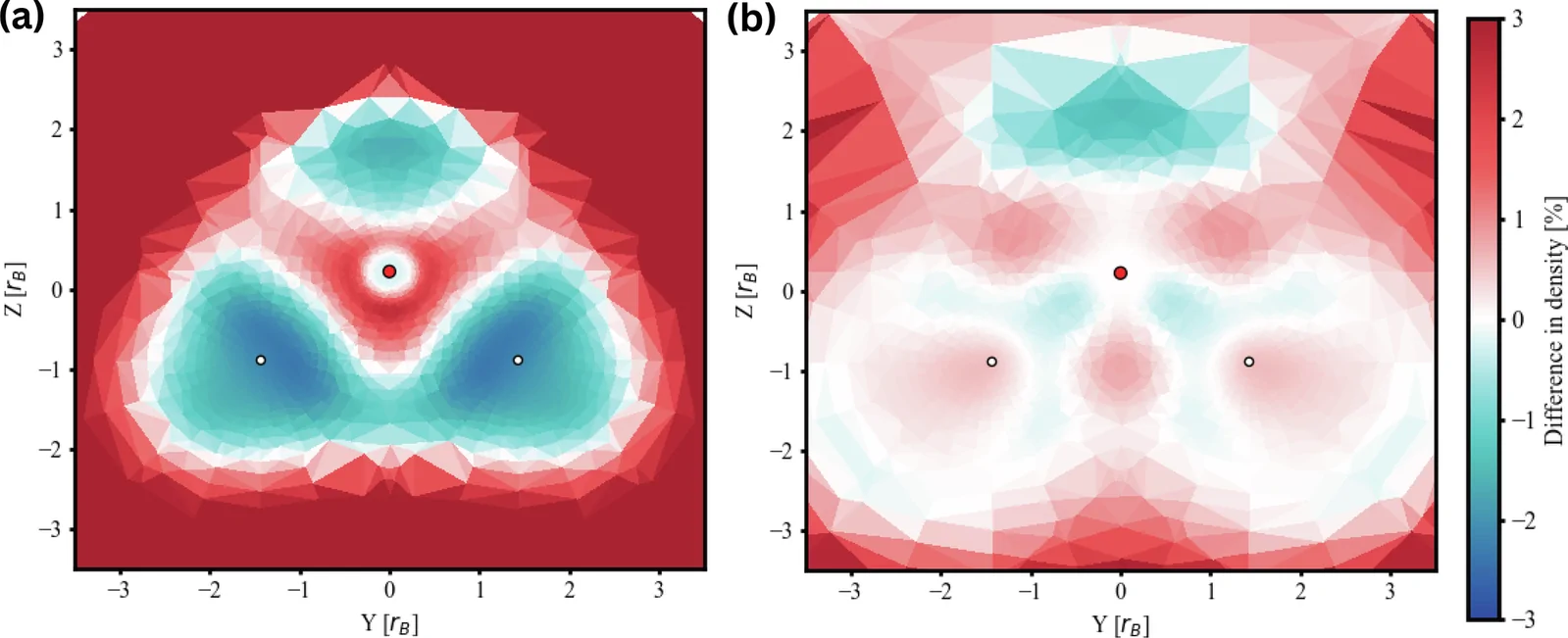
A side-by-side comparison of the electronic density difference w.r.t. CCSD(T) calculations on equilibrium H_2_O. **a** PW-LDA exhibits significant (multiple %) differences in density even in the key bonding regions. **b** Our NN-S LDA predicts densities much closer to the CCSD(T) baseline.

Next, we train an LDA level correction to the PW approximation in the pc-2 basis^41,42^, performing delta learning to optimize a weighted combination of the aforementioned labels with 5 geometries for training and three further ones for validation. Details of data splitting can be found in the section 1 of the supplemental material. The resultant neural network DFA, which we call NN-S, exhibits errors below 1 kcal / mol on all energetic properties across the remaining 112 geometries from the full dataset as seen in Fig. 2. This is notable, particularly in contrast to lower performance from the higher rung PBE and B3LYP functionals chosen for comparison. The improvement in electron densities with respect to PW-LDA is further highlighted in Fig. 3. While this set of comparison functionals is by no means exhaustive, it constitutes a likely selection of models for an unknown application, which would be the key use case for cheap specialist functionals, while covering a range of rungs on Jacob’s ladder. Furthermore, the performance of PBE on the WATER27 dataset used later in this work is remarkably strong for a relatively simple functional. PBE outperforms not only B3LYP, but also dispersion corrected variants of both PBE and B3LYP as well as SCAN on the WATER27 dataset^11^. Additionally, other modern functionals such as PBE0, *ω*-B97X-D3 or the double hybrid DSD-B3LYP have similar median absolute deviations to PBE on the WATER27 dataset, ranging from 2 to 3 kcal / mol^11^.

While the performance on this dataset is interesting, it is not too surprising that we see improvements on properties present in the training set. Indeed, some of the more promising NN DFA approaches published in the past show overfitting to particular properties at least to some extent^23^. However, we may furthermore consider properties outside the training set to assess performance of each DFA. When comparing to experimental results, we find that NN-S still outperforms base model PW-LDA across the board, and that it exceeds PBE performance across all properties but infrared intensities, as seen in the supplementary material. Thus, we can clearly see that NN-S is an improved DFA in the context of systems of single water molecules, and that we can realize an explicit gain by overfitting to this specific chemical context. Crucially, overfitting is the correct term here, and although this is usually considered a negative, it can be beneficial in the STEP paradigm.

As long as the domain we overfit to is broad enough, we can view this simply as an improvement in accuracy, particularly as there is no noise attached to our training data. In our case, we overfit to the chemical context of molecular H_2_O, and we have clear evidence that the resultant DFA is unphysical as shown in the supplementary material. However, the resultant DFA is optimized for H_2_O, indicating that relevant systems are either insensitive to the unphysical characteristics with respect to the properties of interest, or that a distortion in contributed energies from these densities more accurately captures the nature of XC effects in water.

Overall, this overfitting clearly benefits calculations within the context of molecular H_2_O, including improved bond breaking energies and ionization calculations. However, such a narrow scope is not particularly useful, as most interesting behavior in water occurs at the intermolecular level. We thus turn to the WATER27 subset^43^ of the GMTKN55 database^11^, to see how our original model performs on this data, and how gains on single-molecule systems may be transferred to clusters of multiple molecules.

### Transfer learning and application to water clusters

Larger clusters of water molecules exhibit dispersion interactions that are notoriously difficult to capture with density functional theory. Thus, these systems typically warrant dispersion corrections and/or the use of higher rung DFAs, although neither guarantees improved performance. Furthermore, they reach the scale of systems that become prohibitively expensive to model with CCSD(T) calculations. These two challenges make such systems particularly interesting for the application of neural specialist DFAs. However, as such a DFA needs to be retrained on every chemical context it is applied to, training on a large dataset of more extensive systems is undesirable. In an effort to maintain the comparatively low training cost of our single-molecule model, we thus continue with a low data approach and proceed to study how we can obtain accurate predictions on the WATER27 dataset with minimal data and only small systems in the training set.

The cheapest approach is to use the single-molecule NN-S model to extrapolate to multi-molecule systems. While there is no reason to assume that this model will perform well in this extrapolative regime, it is nevertheless instructive to see how it compares at least to PW-LDA when extrapolating to closely related systems. If such an extrapolation leads to significantly worse predictions than the base model, this would restrict specialist functionals only to systems well within their training domain.

Next, we consider the cheapest and easiest way of incorporating some information about multi-molecule effects into our neural DFA. While we could establish a large dataset of two-body and three-body interactions for training similar to^30^, this approach requires substantial datasets which exist for water, but not for other systems where specialist models are of interest. Instead, we use a transfer learning approach to add a small amount of multi-molecule information into our existing NN-S model. We retrain the model on the smallest ${({{{\rm{H}}}}_{2}{{\rm{O}}})}_{2}$ entry of the WATER27 dataset until a specified error threshold of 1 kcal / mol is met. This happens very rapidly, within 20 epochs, due to the fact that we are training on a single scalar value. It was found that any longer training yields only negligible gains on the dimer energy, but significantly decreases the model’s performance on other system. This threshold furthermore aligns with a transition in the training history, with losses decreasing much more rapidly before this point than after. We dub the resultant model NN-T, and evaluate its performance on the remaining entries of the WATER27 dataset.

Finally, we compare these models against a third candidate model, which we call NN-2, which is similarly trained only on the ${({{{\rm{H}}}}_{2}{{\rm{O}}})}_{2}$ entry of the WATER27 dataset, but beginning with a random initialization. This allows us to see whether the transfer learning is required, and whether we could even realize gains by simply learning from a single scalar.

All of these models are trained at pc-2 basis level, and the resultant models vary greatly in their exact functional form. A visual representation of this can be found in the supplementary material. In particular, NN-T retains a lot of features from NN-S, while NN-2 is completely distinct and has an unphysical sign across the entire density range encountered in water molecules. Notably, NN-T and NN-2 have converged to completely different local optima of the LDA space, indicating that transfer learning has had a pronounced effect.

Each model is now evaluated against reference CCSD(T) calculations from the GMTKN55 database. However, as the latter calculations are done in the complete basis set (CBS) limit, we calculate system energies using our three models in the pc-1, pc-2, and pc-3 bases and use the exponential square root Ansatz from Jensen^44^ for extrapolation. We model the energy in the pc-*γ* basis as

2 $${E}_{\gamma }={E}_{CBS}+A\cdot {e}^{-\beta \sqrt{\gamma +1}},$$

with *γ* + 1 in the exponent, as the pc family is zero-indexed. *E*_*CBS*_, *A*, and *β* are parameters specific to each modeled system, which we solve for using the finite basis calculations for a given system. We may then take *E*_*CBS*_ to be the CBS energy of the system.

**Fig. 4:**
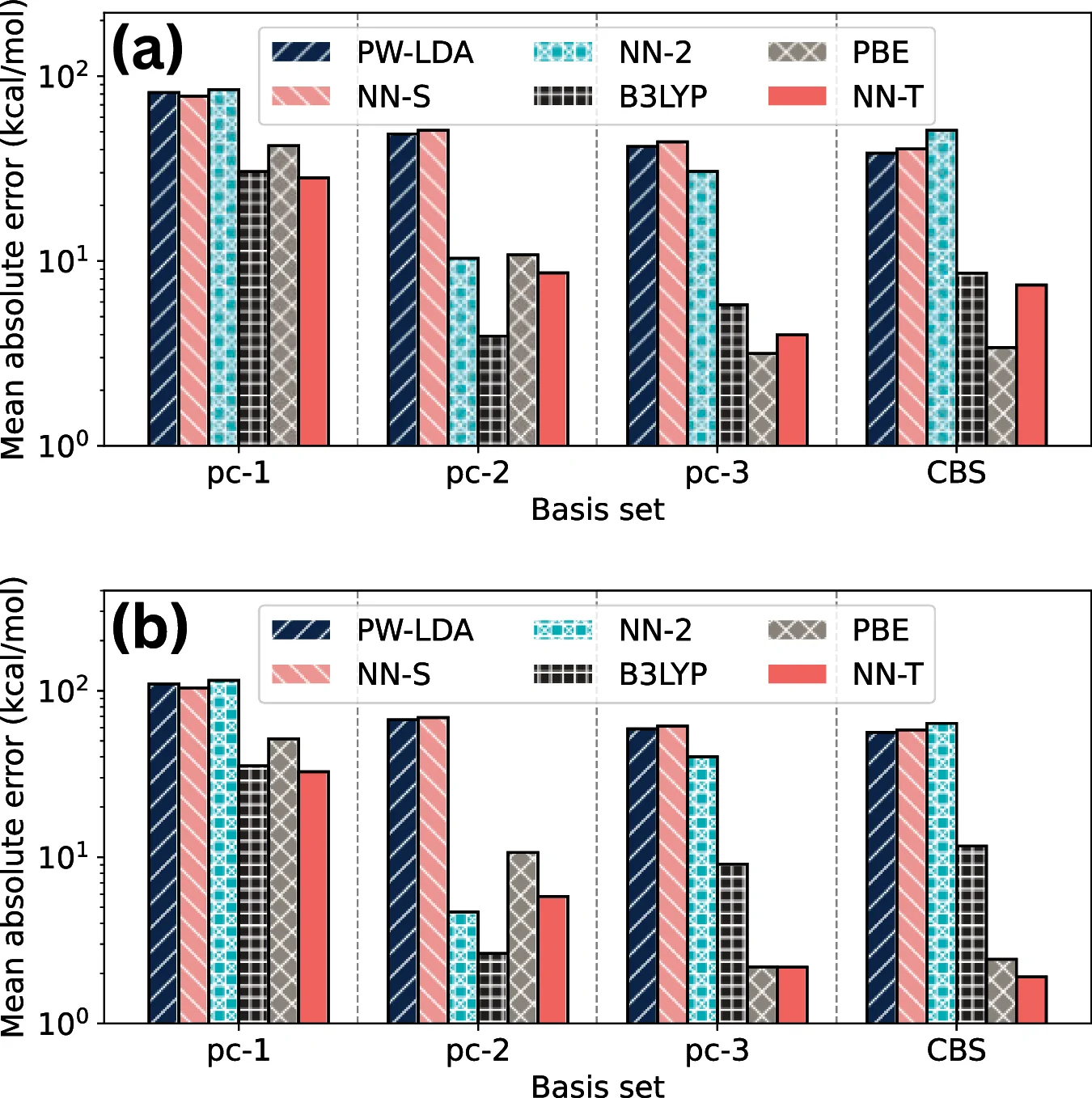
Mean absolute error of different models at different basis set accuracies on the WATER27 subset of GMTKN55 excluding the water dimer used for training of NN-2 and NN-T models. The pc-*γ* are different polarization consistent basis sets, while CBS indicates the complete basis set limit. **a** Vertical bars indicate MAE on the entire remaining WATER27 subset. **b** Vertical bars indicate MAE only on the neutral subset of the remaining WATER27 data.

The resulting mean absolute errors for each model and basis combination across the WATER27 dataset excluding the ${({{{\rm{H}}}}_{2}{{\rm{O}}})}_{2}$ entry which was used for training are shown in Fig. 4a. A few trends emerge quite clearly. For one, the single-molecule NN-S model performs similar to the baseline PW, indicating that it has reasonable performance even in a slightly extrapolative regime. This increases our confidence in neural specialist DFAs as a more general approach, as such a model would be of little use if it failed completely when used even slightly outside the training domain. Secondly, we see that the transfer-learned NN-T model performs remarkably well, being the most accurate model at the pc-1 level, and the second most accurate at the pc-2 through CBS levels, despite having a substantially restricted input space compared to the hybrid B3LYP and the PBE-GGA DFAs. This is a notable result as it indicates that little data can suffice to unlock substantial performance gains in neural specialist DFAs if one does so carefully. The importance of careful training is further emphasized by the poor results from the two-body only NN-2 model, which performs much worse than its transfer-learned counterpart. We see reasonable improvements at the pc-2 level, where the NN-2 model outperforms PBE, but error levels are much higher for all other basis levels, yielding the worst performance of all DFAs under consideration at pc-1 and CBS levels. This behavior is a telltale sign of the DFA compensating for the basis set error, which leads to underestimated energies as the complete basis set is approached, and indicates overfitting to a particular basis level. Similar but weaker effects can be seen for B3LYP, PBE, and NN-T model, with B3LYP exhibiting the strongest effects. PBE errors rise only slightly from the pc-3 level to the CBS limit and interestingly NN-T performs best at pc-3 level despite being trained at pc-2 accuracy.

**Fig. 5:**
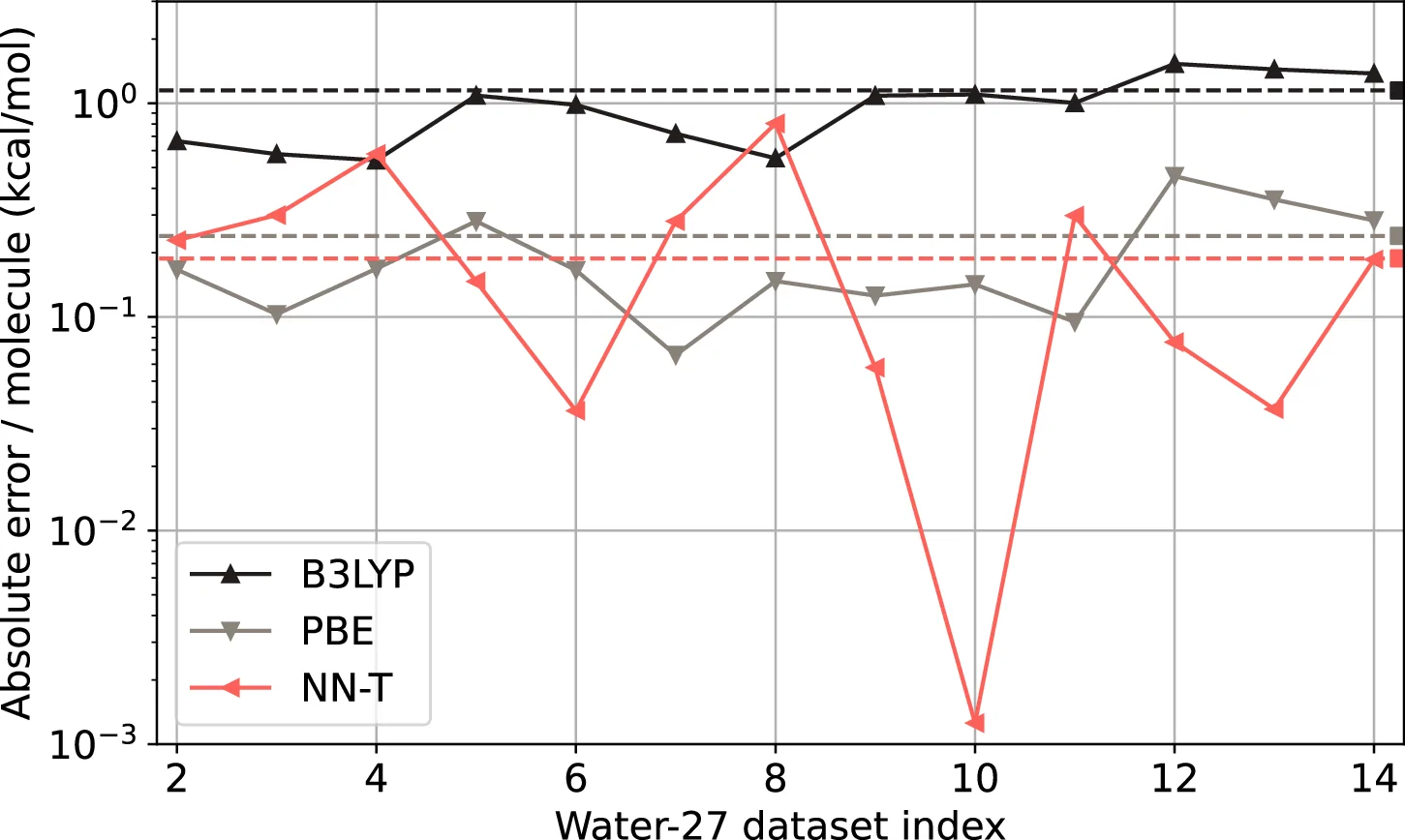
Absolute error per monomer of different models at the CBS limit on the neutral subset of the WATER27 dataset. Solid lines indicate the performance on each individual system, while the dashed lines indicate mean absolute error per molecule.

These differing behaviors are explained in large part by the diverse makeup of the WATER27 dataset. The three main groups contained therein are clusters of neutral water molecules, protonated and deprotonated clusters. Evaluating performance on the neutral entries, which contains clusters of up to twenty molecules, in the CBS limit, we see that the transfer-learned NN-T is not only the best performing model on this dataset at pc-1, pc-3 and CBS levels as shown in Fig. 4b, but that the absolute error per monomer is also stable with system size as seen in Fig. 5. This is a strong indicator of the model extrapolating well to even larger water clusters, in contrast to for example the DM21 model, which was found to have errors increasing with cluster size on a similar water dataset^45^. Additionally, we note that the NN-T model improves monotonically on this subset with increasing basis completeness, and provides strong results across all basis set levels. Although PBE is also close to monotonic improvement, this is an important result as it demonstrates that gains seen are not simply due to cancellation of basis set errors.

The main errors in the CBS limit for NN-T arise for protonated and deprotonated clusters, which is perhaps unsurprising for a model never exposed to any H_3O+_ or OH- ions. Interestingly, the structure of the error differs between protonated systems and deprotonated ones. In the former case, NN-T exhibits underprediction of energies at each basis level with errors increasing with basis set completeness, indicating overcompensation against basis set error. On the latter dataset however, we observe convergence towards the correct energies but with a high rate of convergence, which leads the exponential square root ansatz to underpredict CBS limit energies. We find that this latter issue can be mitigated by choosing a different extrapolation ansatz, as shown by a direct comparison to a mixed exponential scheme^46^ in the supplemental, which achieved much improved NN-T results for OH^−^ containing systems, making it the best performing model on this subset while also resulting in more modest improvements for PBE and B3LYP predictions. Finally, the last entry of the WATER27 dataset exhibits a combination of both effects, as it is the only one to feature both types of ions. Interestingly, the NN-T model here is the only one to approach the true energy with increasing basis accuracy, as all other DFAs investigated significantly underestimate the resulting energy. Ultimately, for this system NN-T exhibits a deprotonated signature while all others exhibit the underprediction characteristic of protonated systems. Similarly to the other deprotonated systems, NN-T with a modified CBS scheme achieves the lowest error across all DFAs under consideration, improving on B3LYP predictions with either CBS scheme by 0.5 kcal / mol. However, the stark dependence of NN-T performance on the CBS scheme nevertheless illustrates that caution must be taken with such heuristic models as their weaker theoretical grounding makes them more susceptible to large differences in performance from choices such as the extrapolation scheme.

**Fig. 6:**
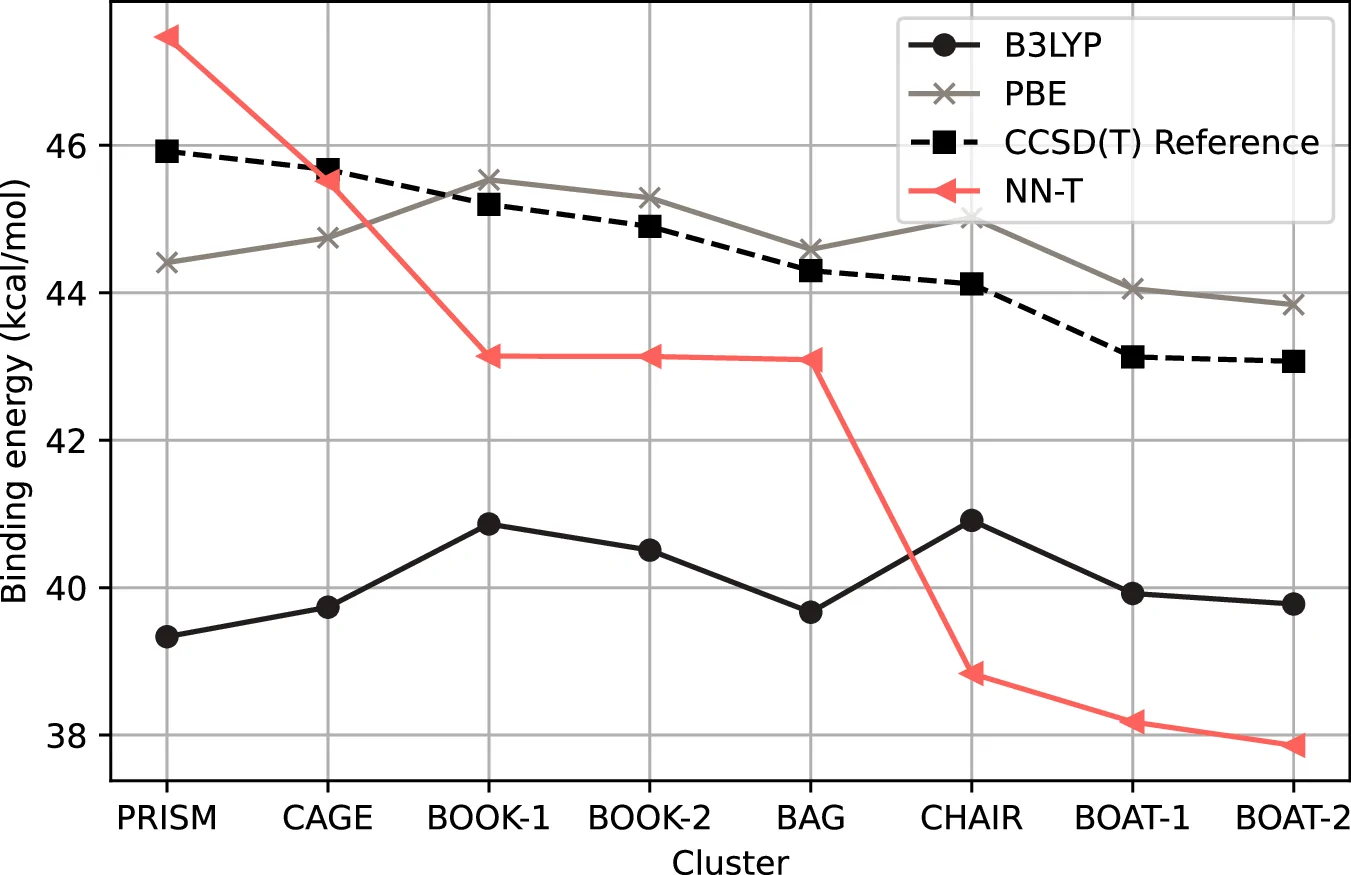
The binding energies of different configurations of ${({{{\rm{H}}}}_{2}{{\rm{O}}})}_{6}$. Black dashed data indicates the CCSD(T) labels, while the solid lines indicate different models. PW-LDA, NN-S, and NN-2 models not shown as their predicted energies are 25 kcal / mol higher than CCSD(T) references for PW-LDA and NN-S, and 30 kcal / mol lower for NN-2.

Next, we compare how well different models characterize the performance of different configurations of the same system, specifically, the energy difference between the eight low-lying hexamer configurations of water taken from Reddy et al.^47^. This question is of particular interest as it constitutes a key factor in the ice phase prediction while constituting a well-known and studied challenge for DFAs^48–50^. The results of our analysis are shown in Fig. 6. We find that NN-T is significantly more accurate on mean absolute deviations of total energies for these systems than B3LYP, PW-LDA, NN-S, and NN-2, while qualitatively capturing the correct energetic ordering of the systems, something both B3LYP and PBE fail to do correctly. However, the absolute errors of the NN-T model remain relatively large, and energy differences between some of the configurations, in particular the BAG-CHAIR pairing, are predicted poorly, with not even NN-S and PW-LDA exhibiting a similarly large energy difference. We conducted an investigation into 2-body and 3-body effects to assess the origins of this error. The results, presented in the supplementary material, show that the NN-T model predicts substantially weaker 2-body and 3-body interactions for these hexamers than reference calculations^47^. This seems to indicate that the bulk of the improved energetics compared to B3LYP and LDA models arises from improved single body calculations with adjustments to two- and three-body calculations balancing the errors across different clusters.

Lastly, we performed some additional analysis to test the performance of the NN-T model. All calculations that follow were conducted in the pc-2 basis with the same configuration as used for training the NN-T model. One interesting question is how much this model retains the performance of the NN-S model on the monomer dataset, and we found that performance is degraded significantly, as expected for a specialist model for cluster energetics and the distinct functional shapes exhibited by both models. In particular, the LEL error is much increased even compared to the LDA model, leading to incorrect total energies and ionization energies on single molecules. Interestingly, the atomization energies are still somewhat improved compared to the PW-LDA baseline, with an MAE of 33.4 kcal / mol, compared to 38.0 kcal / mol. This could be seen to reflect the fact that training on the dimer binding energy focuses more on energy differences, rather than densities or total energies. The full results on total, atomization and ionization energies as well as the LEL are presented in the supplementary material.

Next, we completed similar analysis to the secondary properties discussed for the NN-S model, applying NN-T to calculate the dipole moment of the water dimer contained in WATER27. While this is the same system used for training, only the binding energy of the dimer compared against isolated monomers was used, so the dipole moment is considered a valid property for evaluation. Here, we use the same procedures as used in the supplementary material for calculation of the monomer dipole moment, which was described in detail in ref. ^37^, and find that NN-T predicts a dipole moment in excellent agreement with estimates from experimental measurements^51^ yielding *μ* ≈ 2.63D. The NN-T error is further found to be substantially lower error than all reference functionals on this test case as presented in Table 1.

**Table 1:** Dipole moment *μ* of the dimer and errors by model

Model	Value	Error against ref. ^51^
PW	2.849	0.249
PBE	2.686	0.056
B3LYP	2.890	0.290
NN-T	**2.633**	**0.003**

Bold text indicates lowest error.

**Fig. 7:**
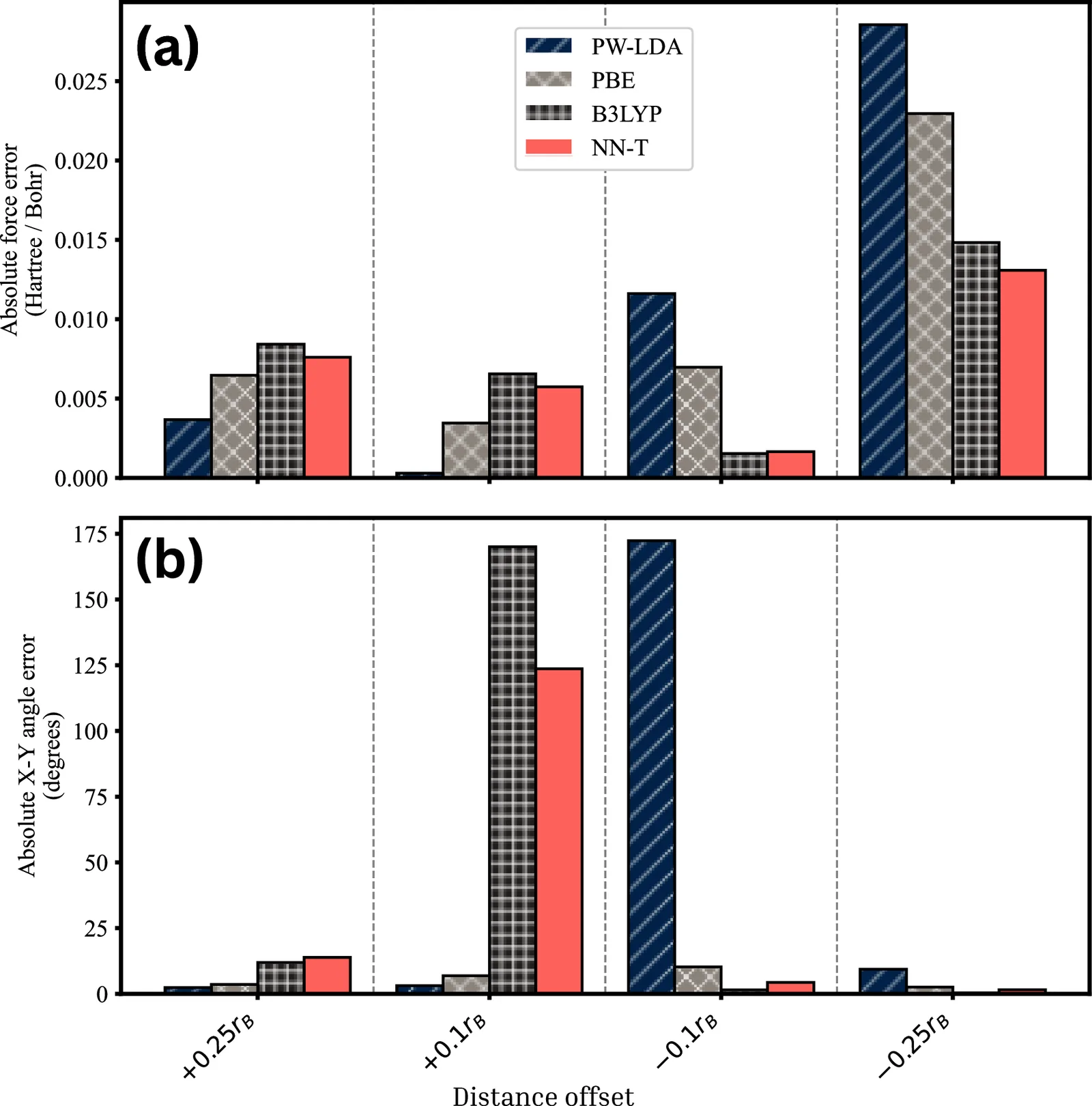
Intermolecular forces in the water dimer compared against MB-pol. X-axis indicates offset in *r*_*B*_ from the optimized MB-pol geometry along the axis connecting the oxygen nuclei. **a** Absolute magnitude error of forces for different models at different separations. **b** Error in the angle of the force.

Finally, we turn to the study of forces on the water dimer, in comparison against MB-pol^47,52–55^ reference forces, to understand whether NN-T is capable of capturing not only energetics, but also intermolecular forces accurately, which become relevant in applications such as molecular dynamics. Here, we take the reference geometry for the dimer in WATER27, optimize it for MB-pol and and change the separation of the oxygen nuclei and associated monomers by ± 0.1*r*_*B*_ and ± 0.25*r*_*B*_. The resulting intermolecular forces are shown in comparison against MB-pol calculations conducted with MBX^56^ in Fig. 7. Here, the NN-T model performs very well on the compressed dimer, achieving the lowest and second-lowest error for the − 0.25*r*_*B*_ and − 0.1*r*_*B*_ separations, respectively, and with only B3LYP achieving a slightly better result on the latter. For longer dimer separations, however, the picture is more mixed. Both B3LYP and NN-T perform poorly for + 0.1*r*_*B*_ separation, substantially underpredicting forces and yielding large errors in angle. This can be accounted for by the fact that both slightly overpredict the equilibrium distance, thus placing this separation closer to their energetic minimum, leading to weaker forces, and more arbitrary directions. The same effect can be observed for PW-LDA at − 0.1*r*_*B*_ separation, as PW-LDA underpredicts the distance significantly. All models recover much more accurate angles and lower relative force errors as we move to the more strongly stretched or compressed regime. However, the NN-T model continues to perform relatively poorly for + 0.25*r*_*B*_, for which PW-LDA is actually found to be the best fitting DFA under consideration, with PBE performing better than NN-T also, and only B3LYP performing worse. Overall then, NN-T performance on forces seems to be limited primarily to shorter intermolecular separations, where the strongest forces between molecules are captured better than by the other DFAs.

## Discussion

While specialist ML-DFAs have been proposed in the past, requirements for large datasets and relatively complex constructions have made them unfeasible as a general scheme for applications to arbitrary systems. Here, we have demonstrated that simple specialist LDA functionals are capable of improving calculations on systems of interest compared to commonly used DFAs on the lower rungs of Jacob’s ladder for the case of water. Our proposed NN-S and NN-T models exhibit good performance on their respective domains, which we demonstrate to be sufficiently large to enable calculations of different properties of interest. However, we also find some clear limitations to the utility of the NN-T model in particular. For one, it struggles with extrapolation to ionized clusters in the WATER27 dataset, specifically exhibiting poor performance in the CBS limit on deprotonated clusters depending on the chosen CBS ansatz. While we demonstrate that this issue can be resolved by choosing a different ansatz, the strong dependence of the model on the choice of CBS scheme is a limitation in its own right, indicating that much care is to be taken with heuristic models in a low data regime. Furthermore, the much improved performance of the NN-T model in particular with respect to PW-LDA does not make this a state-of-the-art model. Errors remain substantially higher than those obtained both by specialist functionals trained on more data and by the best non-empirical DFAs for water. One option to potentially improve performance would be training on augmented basis sets with diffuse functions for better non-covalent modeling, although this typically incurs higher KS convergence and training times. Dispersion correction and the inclusion of further training data provide two alternative routes for improvement, but it remains unclear what performance could be achieved with such changes and more work is required to fully optimize these functionals for usage in calculations on water. Nevertheless, the strong performance against standard functionals with only minimal training data and such a simple computational ansatz demonstrates that specialist DFAs can be trained at low cost, opening the door to apply similar methodologies to a wide range of different systems with relative ease. Potential next steps in this line of research include the enhancements of the NN-T model discussed above, as well as the usage of neural specialist DFAs for different chemical contexts and extended systems, correction for higher-order many-body effects, and applications in ab initio molecular dynamics.

## Methods

### Computational details

Our single molecule calculations are based in the ANI-1ccx dataset^39^. Rather than using the approximate CCSD(T) energies from this dataset, we only use H_2_O geometries contained within. We then use PySCF^57–59^ CCSD(T) calculations with the pc-2 basis set^41^ obtained from basis set exchange^60–62^ and sg3 grid^63^ to obtain energies and CCSD(T) reduced density matrix density distributions across these systems, from which our target properties can be derived.

The multi-molecule geometries and CCSD(T)-CBS energies of the WATER27 dataset^43^ were taken from the online listing of the GMTKN55 database^11^.

### Calculation of molecular properties

For each system *X* from the ANI1-ccx^39^ set of geometries containing atoms *X*_*i*_ where *i* indexes the atoms, we calculate the four single molecule properties as shown in Table 2. These properties were chosen as they are representative of different aspects of the electronic calculation while being trivial to calculate from a converged DFT calculation.

**Table 2:** Summary of molecular property calculations

Property	Formula
Atomization E.	*E*(*X*) − ∑_*i*_*E*(*X*_*i*_)
Ionization E.	*E*(*X*) − *E*(*X*^+^)
Total E.	*E*(*X*)
LEL	See eq. (1)

*E*(*X*) indicates the total energy of system *X* from the DFT calculation.

### The neural density functional approximation

**Fig. 8:**
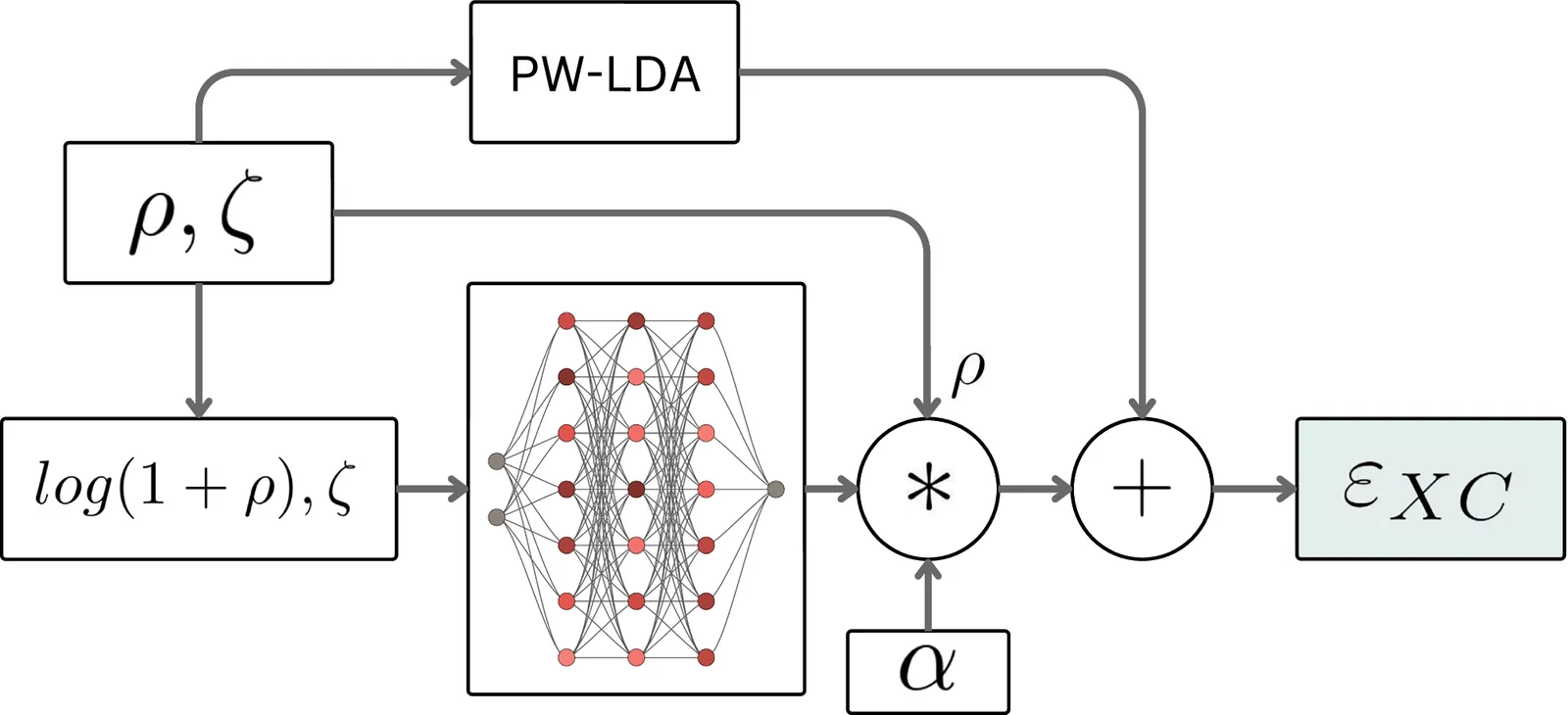
The delta-learning approach used to create the neural network density functional approximations (NN-DFAs). Input density *ρ* and polarization *ζ* are transformed and passed to a neural network, whose output is weighted by a learnable parameter *α*. This term is treated as a per-particle XC energy correction. We multiply it by *ρ* and add it to our base model (in this case PW-LDA) and obtain an overall estimate of XC energy density *ε*_*XC*_.

The neural DFA presented in this work leverages delta learning starting from the spin-polarized PW-LDA, as shown in Fig. 8. A neural network contribution to the local exchange-correlation energy density is calculated as

3 $${\varepsilon }_{XC}^{NN}=\alpha \cdot \rho \cdot f(log(1+\rho ),\zeta ;{\theta }_{NN}),$$

where *ρ* denotes the local electron density and *ζ* indicates spin polarization. The neural network function is denoted as *f* with learnable parameters *θ*_*NN*_. In addition, *α* is a learnable parameter initialized to zero, ensuring smooth transition from the PW-LDA to the neural representation. Thus the full set of learnable parameters is

4 $$\theta =\{{\theta }_{NN},\alpha \}.$$

The input transform *log*(1 + *ρ*) was chosen to reduce the range of inputs from over five orders of magnitude to two. To maintain expressiveness of the output, we multiply the result by the density *ρ*. The parameters *θ* were optimized using the ADAM optimizer with a fixed learning rate of 0.005 and a mean absolute error (L1) loss.

For the neural network itself, a multi-layer perceptron (MLP) architecture was chosen. Here, the activations of a layer **a**_*j*_ are derived from the previous layer **a**_*j*−1_ as

5 $${{{\bf{a}}}}_{j}=\sigma \left({{{\bf{W}}}}_{j}{{{\bf{a}}}}_{j-1}+{{{\bf{b}}}}_{j}\right).$$

Here, **W**_*j*_ is a linear, learnable weight matrix and **b**_*j*_ is a bias vector, while *σ* indicates an element-wise non-linearity. The details of the MLP architecture are shown in Table 3. Increases in neural network size were not found to yield any improvements.

**Table 3:** Neural network hyperparameters used in training

Parameter	Value
# Layers	3
# Neurons per layer	32
*σ*	Softplus

### Materials & correspondence

Materials for this study are available from the corresponding author upon reasonable request. Please contact the corresponding author for any inquiries.

## Supplementary information

Supplementary Information

## Data Availability

The data that support the findings of this study can be found under the https://doi.org/10.6084/m9.figshare.30657497.

## Code availability

The core code this work is based on can be found under https://www.github.com/OxfordHED/dqc.git.
