# Supplementary material for: Overfitting by design: neural network density functionals for water
Source: NPJ Comput Mater. 2026 Jul 16;12(1):377. doi: 10.1038/s41524-026-02224-5 (PMC13745280; doi:10.1038/s41524-026-02224-5)
Supplement: Supplementary file 1 [file 41524_2026_2224_MOESM1_ESM.pdf]

Supplemental material for "Overfitting by design: neural network density functionals for water"

Karim K. Alaa El-Din[1*], Antonius v. Strachwitz[1], Ana Coutinho Dutra[1], Sam M. Vinko[1]

[1*]Department of Physics, University of Oxford, Clarendon Laboratory, Parks Road, Oxford OX1 3PU, UK.

*Corresponding author(s). E-mail(s): karim.alaael-din@physics.ox.ac.uk;

Datasets

The ANI1-ccx [1] dataset used in this work was constructed iteratively from the ANI1-x dataset, by first selecting 30 random conformations of H_2O from the deliberately diverse ANI1-x dataset, training an approximate coupled cluster potential on this dataset, and adding further data which is explicitly dissimilar from the current set of samples. An iterative approach based on this procedure was used until 120 conformations of molecular water were obtained, which are illustrated along their normal modes in fig. S1. In our work, we use the first 5 entries of the ANI1-ccx dataset for molecular water as training set and the next three as a validation set, implying that 22 of the test set conformations are randomly biased with respect to the training and validation sets, while the remainder is biased away from the training and validation sets as it was explicitly chosen to be dissimilar. We thus use a test set that is at least as diverse as a randomly selected one.

Deviations from PW-LDA

We can see that the single molecule DFA is unphysical by plotting the energy density contributed by our model across different electron densities at zero spin polarization as a percentage deviation from the generally used PW-LDA seen in figure S2. The large fluctuations at or above electron densities of 2-3 r_B^{-3} are unphysical in nature, and would impact any calculations on systems sensitive to this regime. However, despite its unphysical nature, this DFA best captures the electronic behaviour in the single molecule case. Interestingly, the multi-molecule NN-T model retains the qualitative

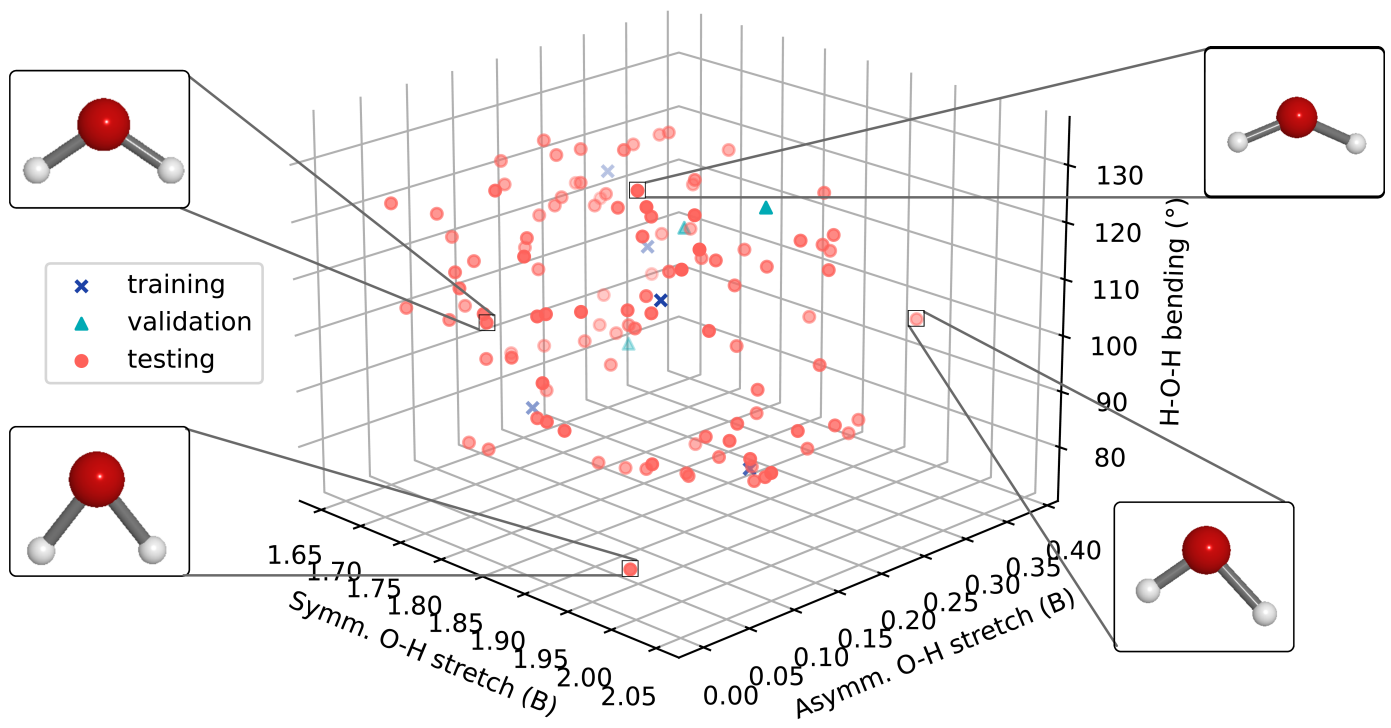

Fig. S1 The dataset of different H_2O configurations characterized by their normal modes. Symmetric stretch, Antisymmetric stretch, and the $H - O - H$ bending angle are shown on the X, Y, and Z axes respectively. Particularly extreme systems are highlighted and their geometries shown. Training data is indicated by blue crosses, while validation is shown as cyan triangles, and testing data is represented as orange circles. Note that only 8 data points out of 120 contribute to training and validation sets.

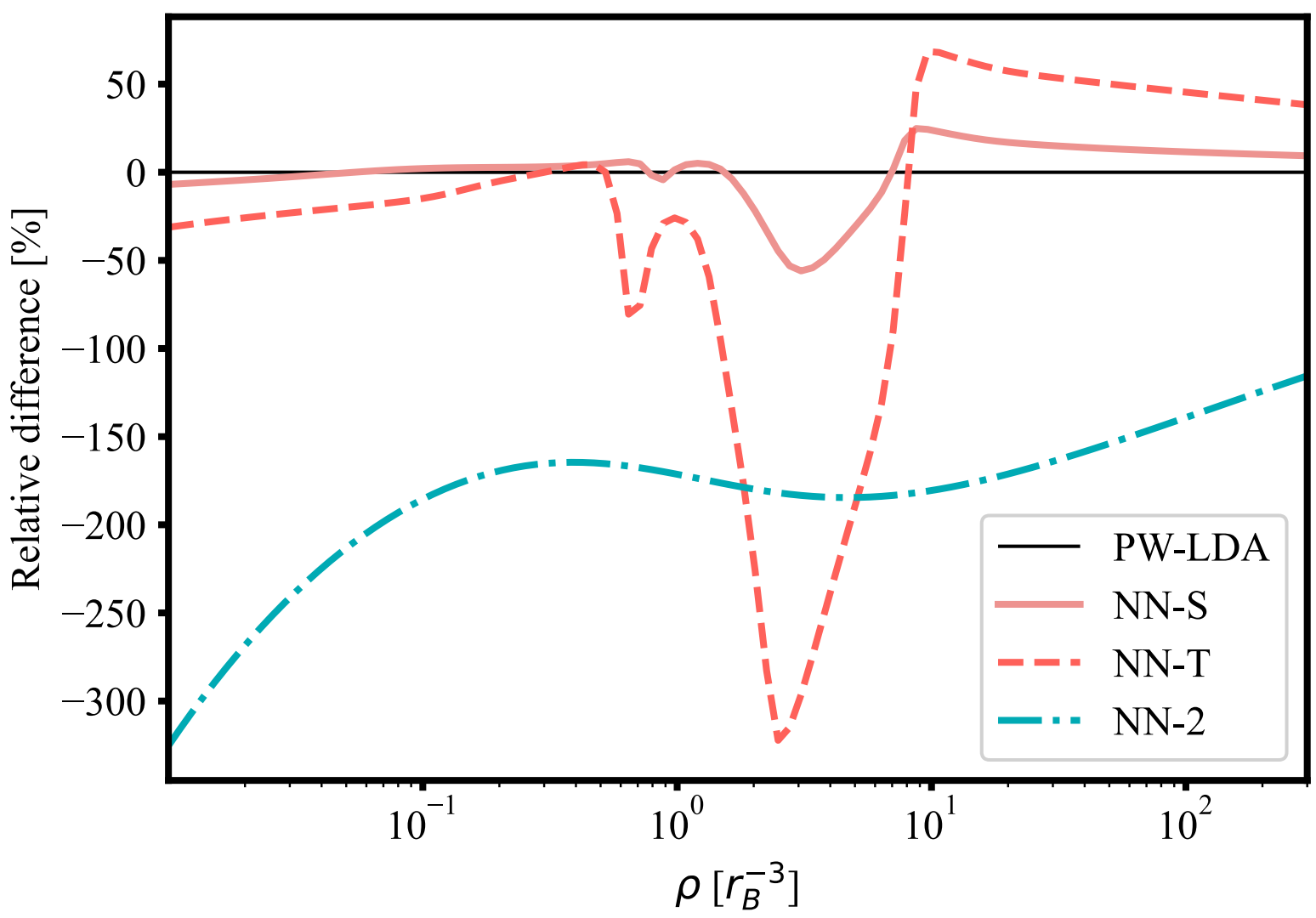

Fig. S2 A comparison of the density-energy density map of the three NN-LDAs considered for intermolecular interaction, and the PW-LDA functional at zero spin polarization.

shape of the NN-S model after transfer learning, with significantly larger deviations. The direct machine learning approach on the two-body system, called NN-2, exhibits a fundamentally different shape, indicating that the NN-T model reaches a different optimum compared to NN-2.

Protonated and deprotonated clusters in the complete basis set limit

The error of the NN-T model on the WATER27 dataset in the CBS limit is primarily driven by the error on the protonated and deprotonated water clusters. Interestingly, the behaviour exhibited by different models on these systems differs depending on whether the cluster contains a protonated H_3O^+ monomer or a deprotonated OH^- monomer as discussed in the main text. Here, we illustrate the difference between the two CBS extrapolation behaviours by considering the protonated and deprotonated dimer entries as shown in figure S3(a) and (b) respectively. In the former case, we see stable convergence to an incorrect value, while the latter demonstrates reasonable convergence for finite basis sets but results in the exponential square root approach yielding very low CBS limit energies. Interestingly, the final entry in the dataset, which contains both a protonated and a deprotonated monomer, yields different behaviour for the NN-T model with respect to the PBE and B3LYP DFAs as seen in figure S3(c). The former approximates the behaviour of the deprotonated systems, while the latter two closer resemble the signature of a protonated system with significant underestimation.

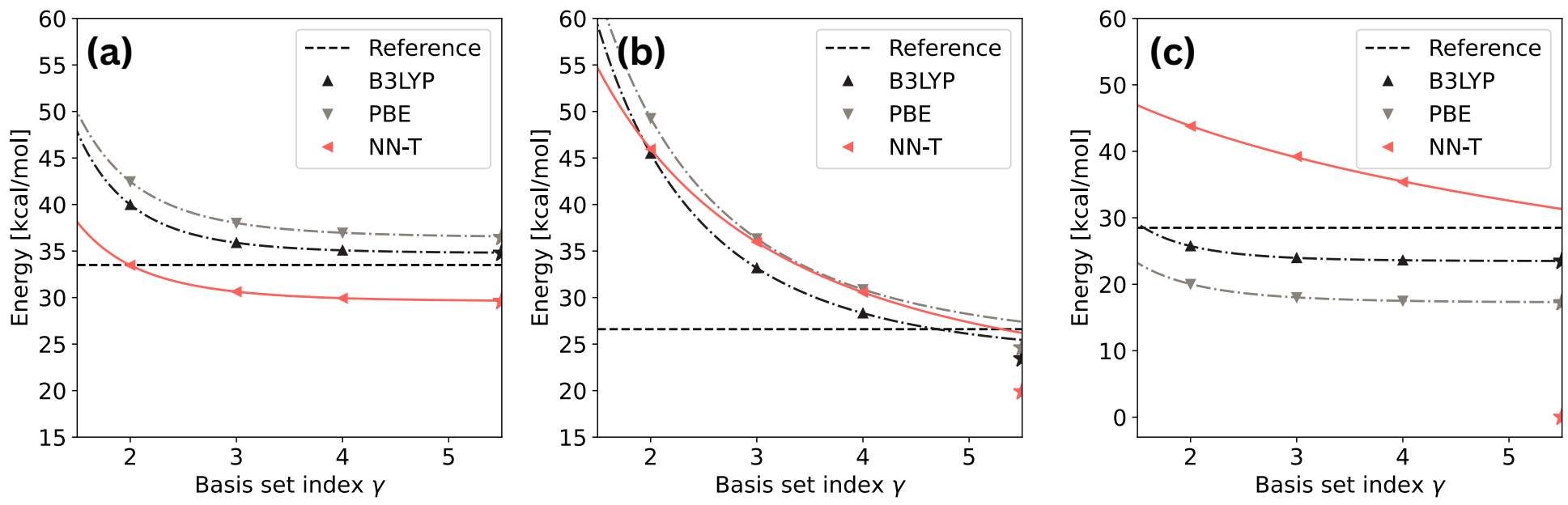

Fig. S3 CBS convergence across different charged entries of the WATER27 dataset. (a) CBS convergence of the protonated dimer entry of the WATER27 dataset shown three different models. The reference is the CCSD(T)-CBS calculation from the GMTKN55 database. (b) Same as (a) but for the deprotonated dimer. (c) Same as (a) and (b) but for the final proton transfer entry of the dataset.

Table S1 Comparison of different CBS extrapolation schemes across the WATER27 dataset excluding only the dimer, and the systems which contain OH^-. Best value for each model across different schemes highlighted in bold.

DFA	Exp. scheme	Mixed scheme
	All entries	
PW	38.32	**38.02**
PBE	3.40	**3.05**
B3LYP	**8.59**	8.75
NN-T	7.41	**3.91**
	All entries containing OH^-	
PW	**18.96**	20.08
PBE	5.76	**3.91**
B3LYP	7.71	**6.54**
NN-T	15.40	**3.32**

Alternative CBS scheme

The exponential CBS extrapolation scheme used for the analysis throughout this paper was found to yield poor results for deprotonated clusters and entry 27 of the WATER27 dataset. This effect can be seen for all models in the case of deprotonated clusters, but only for NN-T on the latter system, due to the qualitative differences discussed in the main text. The stark differences in performance on any finite basis to the CBS limit here led us to conduct additional analysis into alternative CBS approaches. Specifically, the mixed exponential CBS scheme from ref. [2] was found to improve predictions on these systems substantially for the NN-T model with, on aggregate, minor improvements on the other model results. Detailed differences in results are presented in table S1. Conveniently, it also has a closed form expression for the CBS energies which we use, namely

$$E_{\text{CBS}} = \frac{(1+e^2)E_2 - (e+e^3+e^5)E_3 + e^6 E_4}{(e-1)(e^5-e^2-1)}, \tag{S1}$$

where E_2 through E_4 are the energies at the $\gamma+1$ basis set levels. Given the structural differences observed between the NN-T moodel and the other DFAs on the deprotonated clusters, the mixed exponential scheme's increased flexibility in the exact shape of the extrapolation is the most likely cause of this improvement.

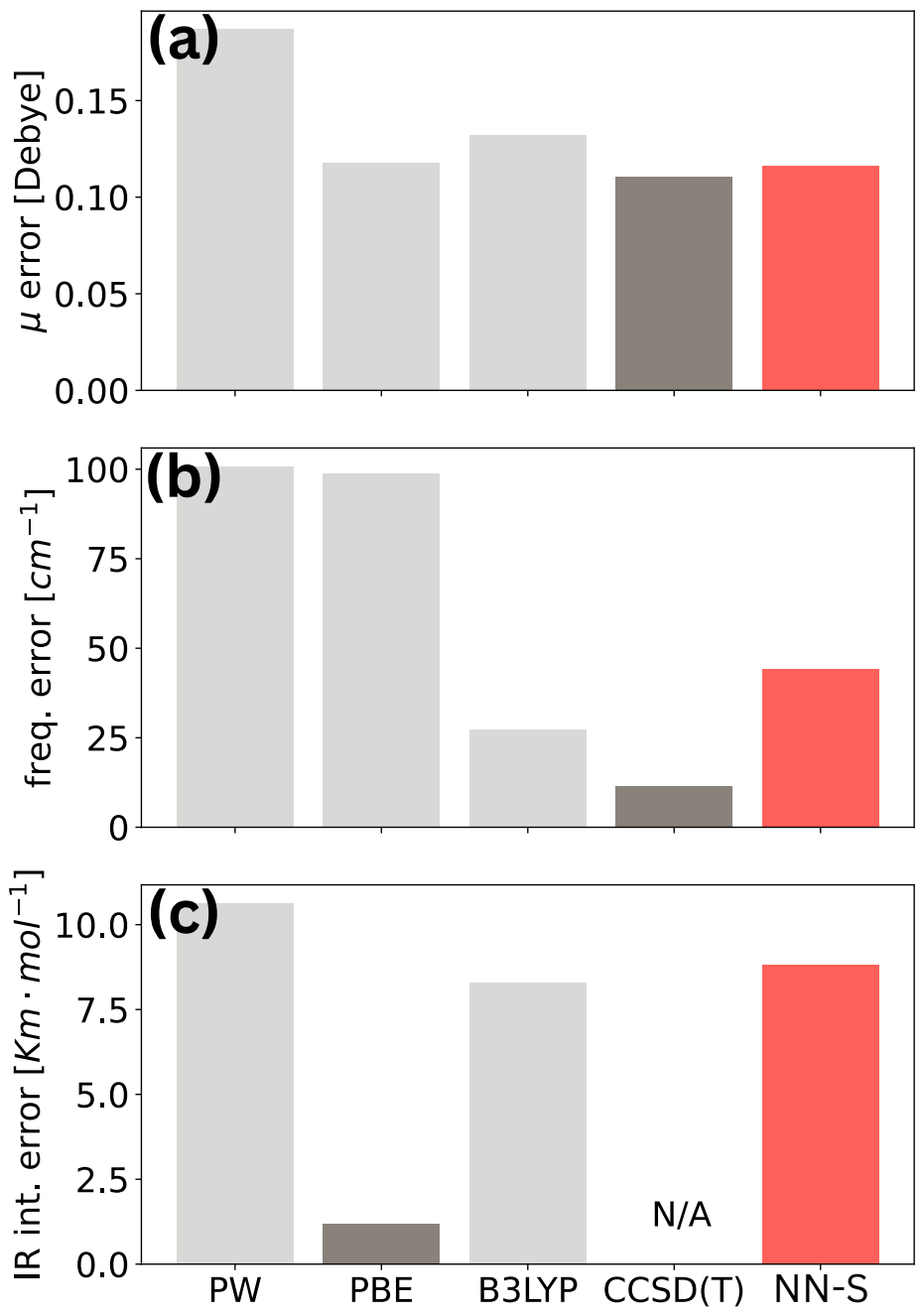

Fig. S4 Predictions of secondary properties from different DFAs and their errors with respect to experimental data. Best model highlighted in dark grey, with the NN-S model (ours) shown in orange. (a) Error in the dipole moment of H_2O. (b) Error in the vibrational frequencies via infrared (IR) spectroscopy. (c) Error in the intensities of the infrared spectral lines.

Derived molecular properties

The properties shown in figure S4 are calculated following the methodology described in Kasim *et al.* [3] from quantum chemistry calculations. Note that the geometry was optimized for each system before calculating dipole moments and infrared spectra.

Application of NN-T to the monomer dataset

Inference on the NN-S dataset was conducted with the NN-T model, as discussed in the main text. The resulting errors were typically larger than the PW-LDA errors due to the large differences in functional form shown in figure S2 of this supplemental, with the exception of the atomization error. The full results are shown in table S2.

Table S2 Mean absolute error of NN-T on monomer dataset.

Property	MAE
Atomization E.	33.4
Ionization E.	32.9
Total E.	508.3
LEL	512.4

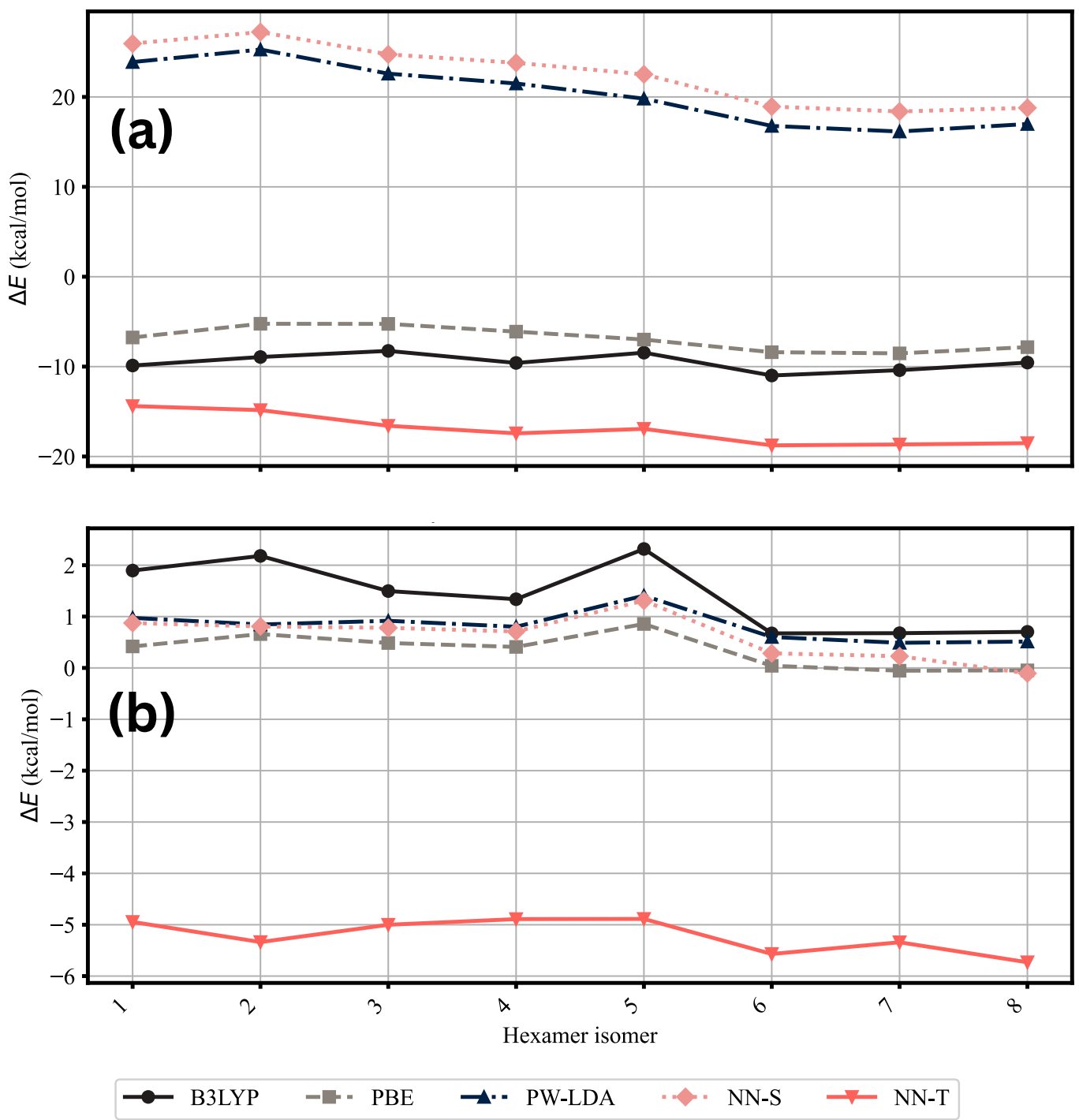

Fig. S5 Errors in many-body effect size with respect to CCSD(T) reference data [5]. All calculations were conducted in the CBS limit for different models on the low-lying hexamer dataset. The NN-2 model was excluded as the CBS energetics were too far from the ground truth CCSD(T) data to be included in the figure. (a) Two-body effect errors. (b) Three-body effect errors.

Many-body effects on the low-lying hexamers

The many-body contributions of the low-lying hexamer dataset were calculated following Dasgupta *et al.* [4] with geometries and reference data from Reddy *et al.* [5]. Results are shown in figure S5. For the NN-S model, the many-body contributions closely track the PW-LDA data, indicating only minimal difference between the two for many-body effects. The NN-T model on the other hand exhibits 2-body effects much closer to the PBE and B3LYP DFAs, all of which underestimate 2-body effects. However, the NN-T model does so with the largest deviation from the CCSD(T)-CBS reference [5]. Furthermore, the three-body effects are substantially suppressed in the NN-T model, not only with regard to the ground truth, but also all other models in this study. Overall, this seems to indicate that the NN-T model achieves the energetic performance seen in the main text by increased single-body deformation (and potentially four-body and above) energies and decreased many body contributions. Unfortunately no many-body reference data exists for the Water-27 dataset to observe these effects in the dataset where NN-T performs best.

References

[1] Smith, J.S., Zubatyuk, R., Nebgen, B., Lubbers, N., Barros, K., Roitberg, A.E., Isayev, O., Tretiak, S.: The ANI-1ccx and ANI-1x data sets, coupled-cluster and density functional theory properties for molecules. Scientific Data 2020 7:1 **7**(1), 1–10 (2020) https://doi.org/10.1038/s41597-020-0473-z

[2] Peterson, K.A., Woon, D.E., Dunning, T.H.: Benchmark calculations with correlated molecular wave functions. IV. The classical barrier height of the H+H2→H2+H reaction. The Journal of Chemical Physics **100**(10), 7410–7415 (1994) https://doi.org/10.1063/1.466884

[3] Kasim, M.F., Lehtola, S., Vinko, S.M.: DQC: A Python program package for differentiable quantum chemistry. Journal of Chemical Physics **156**(8), 84801 (2022) https://doi.org/10.1063/5.0076202

[4] Dasgupta, S., Lambros, E., Perdew, J.P., Paesani, F.: Elevating density functional theory to chemical accuracy for water simulations through a density-corrected many-body formalism. Nature Communications 2021 12:1 **12**(1), 6359 (2021) https://doi.org/10.1038/s41467-021-26618-9

[5] Reddy, S.K., Straight, S.C., Bajaj, P., Huy Pham, C., Riera, M., Moberg, D.R., Morales, M.A., Knight, C., Götz, A.W., Paesani, F.: On the accuracy of the MB-pol many-body potential for water: Interaction energies, vibrational frequencies, and classical thermodynamic and dynamical properties from clusters to liquid water and ice. Journal of Chemical Physics **145**(19) (2016) https://doi.org/10.1063/1.4967719/932964
